# Translation as a regulatory hub for secondary metabolism in *Streptomyces* spp.

**DOI:** 10.1042/EBC20250018

**Published:** 2026-08-19

**Authors:** Amelia Brave, Paul D. Straight

**Affiliations:** 1 Texas A&M AgriLife Research, College Station, TX 77843, U.S.A.; 2 Biochemistry & Biophysics Department, Genetics and Genomics Program, Texas A&M University, College Station, TX 77843, U.S.A.

**Keywords:** antibiotics, biosynthetic gene cluster, natural products, ribosomes, secondary metabolism, translation

## Abstract

Streptomycetes are known for their production of diverse secondary metabolites, many of which have medicinal applications. The enzymes facilitating the production of these metabolites are encoded in biosynthetic gene clusters (BGCs) with regulatory and transport proteins. Regulation of secondary metabolite biosynthesis is tightly controlled in laboratory conditions, and many natural products have low yields or are not expressed in laboratory cultures. Although transcriptional regulation has been a major focus in the past, translational control has recently emerged as a critical determinant of BGC activity. Due to the energy requirement for translation, evidence suggests a greater degree of translational regulation than transcriptional regulation. Many translation factors impact protein synthesis rates, such as regulatory RNAs, ribonucleases, transcript quality control, ribosome rescue systems, tRNA pools, and antibiotic-induced ribosome mutations. These convergent components respond to environmental cues and metabolic states to coordinate secondary metabolism with demands for growth and development of streptomycetes. A deeper understanding of translational control will not only reveal key functions that control and coordinate specialized metabolism but will also highlight new strategies for activating silent gene clusters and enhancing natural product yields. The present review explores how translation serves as a central regulatory hub for BGC activity and discusses emerging tools to manipulate protein synthesis for natural product discovery and biotechnological advancement.

## Introduction

**Figure 1:**
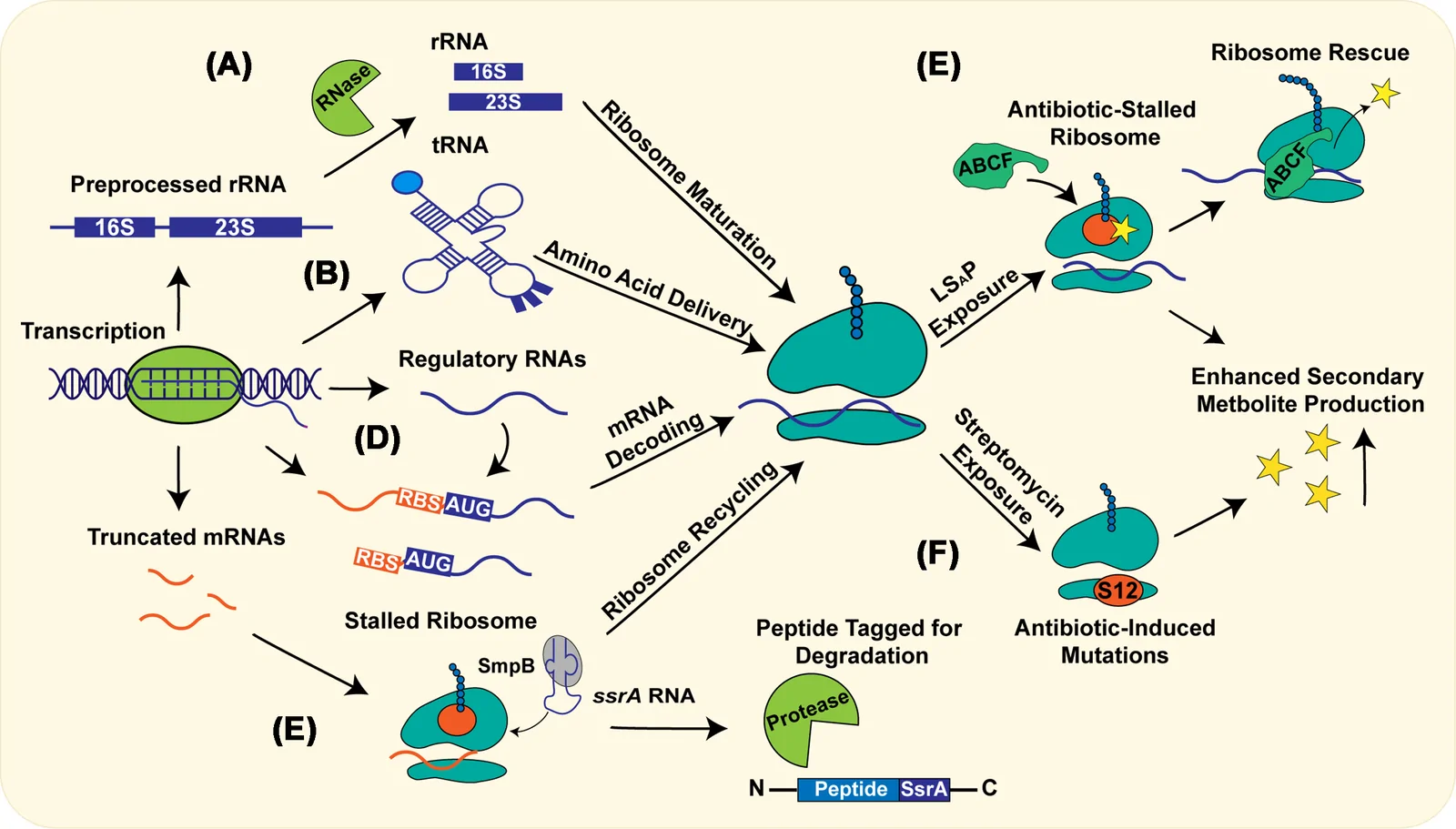
Overview of translation as a regulatory hub Protein synthesis is the sum of multiple regulatory and processing steps. (**A**) Translation machinery is encoded within the genome. RNA polymerase transcribes rRNA, which is further processed by RNases and incorporated into mature ribosomes. (**B**) tRNA is transcribed by RNA polymerase and delivers amino acids to growing nascent peptides at the ribosome. (**C**) Open reading frames (ORFs) are transcribed and regulated by cis and trans noncoding RNAs (ncRNAs) and decoded by ribosomes. (**D**) Truncated RNAs with ORFs lacking stop codons stall ribosomes and require rescue by the transfer messenger RNA (tmRNA)–small protein B (SmpB) system, which tags defective peptides for degradation and recycles ribosomes. (**E**) Antibiotic stalling of ribosomes by lincosamides, streptogramin A, and pleuromutilin (LS_A_P) antibiotics recruits antibiotic resistance (ARE) ABCF proteins, which displace the drug to resume translation and stimulate secondary metabolite production. (**F**) Streptomycin exposure induces mutations in ribosomal proteins that trigger the activation and enhancement of antibiotic production.

*Streptomyces* species have been studied for decades due to their production of antibiotics, antitumor drugs, immunosuppressants, and other bioactive compounds [1]. These medically useful chemicals are produced through *Streptomyces* secondary metabolism. In general, secondary metabolites are unnecessary for the organism’s survival in laboratory cultures but instead offer a competitive edge within its environment. Secondary metabolites are encoded by biosynthetic gene clusters (BGCs) in *Streptomyces* genomes as groups of genes responsible for the regulation, biosynthesis, and transport of product metabolites. Because of the potential to use streptomycetes to produce useful chemicals, it is important to understand regulatory elements controlling BGC expression. In laboratory settings, many BGCs are expressed at low levels or are ‘silent’ and do not produce metabolites [2]. The regulatory elements that stimulate the production of secondary metabolism can be manipulated to overcome low expression levels. Regulation of BGC expression has been extensively studied at the transcriptional level, and key transcription factors have been identified for many BGCs. For translation, studies have focused mostly on methods for ribosome engineering to manipulate metabolite expression. Many studies reveal that antibiotic-induced mutations in ribosomal protein-encoding genes, and in RNA polymerase genes, stimulate antibiotic production in streptomycetes [3–11]. Recently, studies have focused on evaluating post-transcriptional control of translation efficiency that sustains the synthesis of biosynthetic enzymes and the regulatory proteins required for secondary metabolism. Translation efficiency is especially important for biosynthetic enzyme expression because their exceptionally long transcripts are prone to truncation, which stalls ribosomes and reduces pools of translation machinery. Because translation is the most energetically costly step of gene expression, it is advantageous for streptomycetes to have stringent regulation at the translational level to avoid errant expression [12]. As demonstrated for the model organism, *Streptomyces coelicolor*, translation efficiency is enhanced and secondary metabolism most active during the transition from mid-exponential to late stage growth, yet transcription levels remain relatively unchanged [13]. This observation invokes the phenomenon of ‘translational buffering,’ where transcripts occupying ribosomes are more tightly regulated than transcript abundance [14]. The stoichiometry of protein subunits is also maintained at the translation level rather than at the transcription level [15]. Together, these data suggest that the onset of secondary metabolism may be heavily influenced by translation efficiency. Protein synthesis relies on multiple levels of post-transcriptional regulation, through regulatory RNAs, ribonucleases (RNases), tRNA abundance, and proteins to aid in translational efficiency (Figure 1). Protein synthesis sits at a regulatory crossroads involving integration of these regulatory elements and influence from cellular metabolic states and environmental cues. This convergence of translation machinery and external influence can be described as a translational ‘hub.’ In the present essay, we will present instances in which translational components have been identified and some engineered to enhance natural product production in streptomycetes.

## Translational buffering mediated by noncoding RNAs

The transcripts occupying actively translating ribosomes are influenced by regulatory ncRNAs. There are many ncRNA species that influence transcript stability and regulation in various ways. Small regulatory RNAs (sRNAs) regulate expression through affecting mRNA transcript stability and ability to be translated (Figure 1C). Global analysis of sRNAs present in the transition from exponential to stationary phase in *S. coelicolor* identified 63 putative sRNAs and verified expression of 11, suggesting the role of sRNAs in regulating growth and onset of secondary metabolism [16]. For *S. coelicolor*, overexpression of a 6S-like RNA transcript, *scr3559*, enhances production of antibiotics actinorhodin and undecylprodigiosin as well as other nonantibiotic secondary metabolites [17]. The absence of *scr3559* negatively impacts growth and secondary metabolite production [18], likely through inhibition of ribosome biogenesis as seen in *Escherichia coli* [19].

Cis-antisense RNAs (asRNAs) are a type of sRNA that are expressed from the strand complementary to their target protein-encoding gene [20]. Cis-asRNAs are abundant in streptomycetes [21]. Moody et al. discovered a distinct class of asRNAs present as regulatory elements in many streptomycete BGCs. These convergent and untranslated RNAs (cutoRNAs) occur at the overlap of a 3′UTR and a convergently encoded gene. There are cutoRNAs encoded within the actinorhodin and coelichelin BGCs of *S. coelicolor*, the chloramphenicol BGC of *Streptomyces venezuelae*, and the avermectin BGC of *Streptomyces avermitilis* [21]. Though the regulatory implications of asRNAs and cutoRNAs have not been fully evaluated, their presence in many BGCs across several species implies their importance in BGC regulation. It was proposed that these regulatory elements interact with RNase III to bind and/or cleave double stranded segments of transcripts paired with cutoRNA synthesized from the overlapping transcribed regions. This interaction could impact transcript stability and its ability to be translated [21]. Overall, ncRNAs are abundant regulatory elements present throughout streptomycete secondary metabolism; however, their role in transcript regulation remains poorly understood. There is evidence that sRNAs impact expression of translation machinery, which impacts global expression. Further studies on the impact of these and other ncRNAs offer potential for manipulation to enhance secondary metabolite production.

Untranslated regions upstream of ORFs impact translational efficiency. The 5′ untranslated region (5′UTR) contains the ribosome binding site (RBS), the Shine–Dalgarno sequence [22], and structural features that tune translational output. A study on translation efficiency in *S. coelicolor* demonstrated that the length of a 5′UTR impacts the translation efficiency and expression timing of a transcript [13]. Two categories described for 5′UTRs present in *S. coelicolor* transcripts are umRNAs (≥9 nucleotides) and leaderless lmRNAs (<9 nucleotides). Differences in BGC expression are correlated with the type of 5′UTRs. Across all growth phases in streptomycetes, umRNA transcripts are expressed consistently. In contrast, the translation efficiency of lmRNA transcripts increases throughout the streptomycete life cycle and has the greatest efficiency during stationary phase. The lmRNAs are likely to share the same regulatory control, while length and nucleotide content of the umRNA define variable translation efficiencies. In the latter, the differences in free energy of the 5′UTR secondary structures with high GC content may dictate specific mRNA translation efficiencies [13]. Thus, engineering of 5′UTRs in transcripts encoding biosynthetic genes could be utilized to modify secondary metabolism activity.

## RNase influence on antibiotic biosynthesis

As with regulatory RNAs, RNases play important roles in transcript stability and the biogenesis of translation machinery. These enzymes are essential for post-transcriptional regulation in bacteria and contribute to RNA degradation, maturation, and stability [23]. In addition to regulating transcripts, RNases are paramount in the maturation of other RNAs present in translational machinery and post-transcriptional regulation including rRNAs, tRNAs, and sRNAs. Mutants lacking RNases produce more translationally inactive ribosome dimers, 100S ribosomes [24]. RNases are integral global regulators of streptomycetes life cycles and secondary metabolite expression.

Streptomycetes encode multiple RNases that influence antibiotic biosynthesis across species [25]. In *S. coelicolor*, the RNase E ortholog RNase ES is nonessential. Null mutations of RNase ES itself have not been studied for their influence on antibiotic production. However, knockouts of RNase E inhibitor RraAS1 result in accelerated growth and overproduction of actinorhodin and undecylprodigiosin, two *S. coelicolor* antibiotics. The phenotype indicates that RNase ES activity likely suppresses secondary metabolite production [26]. Thus, RNase ES mutations may be beneficial in engineering host strains for BGC expression. RNases have different similar regulatory roles across species. RNase III is a double-strand-specific endonuclease also found in *S. coelicolor*. *Streptomyces coelicolor* mutants lacking RNase III (Δ*absB*) show reduced production of antibiotics [27]. RNase III is required for actinomycin biosynthesis in *Streptomyces antibioticus* [28]. RNase J, a 5′-to-3′ exonuclease, is also nonessential yet has differential impacts on specific secondary metabolites from different organisms. Loss of RNase J reduces calcium-dependent antibiotic production while increasing undecylprodigiosin levels in *S. coelicolor* [29]. RNase J deletion in *Streptomyces lincolnensis* dramatically increases production of the protein synthesis inhibitor lincomycin [30]. Null RNase J mutations in *S. venezuelae* reduce production of the antibiotic jadomycin; however, transcript abundance of biosynthetic genes *jadA* and *jadM* are unchanged [24]. Therefore, in *S. venezuelae* RNase J can regulate antibiotic production through a mechanism other than mRNA degradation.

Ribosome biogenesis relies on the maturation of several rRNA species. This maturation is mediated in bacteria by various RNases (Figure 1A). Deficiencies in ribosome biogenesis due to the absence of RNases negatively impact global antibiotic production. *Streptomyces venezuelae* requires RNase J and RNase III for preprocessing of rRNA required for ribosome maturation. The deletion of RNase III and RNase J yields an abundance of hibernating 100S ribosome dimers [24], which is seen in *E. coli* primarily during stationary phase as metabolic rates decrease [31]. Ribosome dimers are considered translationally inactive due to blockages in the peptidyl transferase center and exit channels and their lack of association with transcripts and tRNAs [32–34]. This defect in ribosome assembly in addition to the abundance of large transcripts required to translate modular enzymes common in BGCs likely leads to decreased production of antibiotics in streptomycete mutants lacking important RNases.

## Quality control of large biosynthetic transcripts

The biosynthesis of many secondary metabolites requires large, multi-modular enzymes including non-ribosomal peptide synthetases and polyketide synthases (PKSs). These genes often exceed 10 kB in length with equally massive mRNA transcripts. These long transcripts are especially prone to truncation caused by premature transcription termination or degradation by RNases [35–37]. Because transcription and translation are coupled in bacteria, truncated transcripts are translated into polypeptides. Polypeptides translated from truncated ORFs are unable to facilitate metabolite biosynthesis and can be toxic to cells [38]. Thus, translation of these catalytically inactive polypeptides is not only a waste of cellular resources but may be a hazard. Truncated mRNAs with ORFs lacking stop codons stall ribosomes, which renders ribosomes unusable, reducing translational efficiency within a cell [39]. To overcome this challenge, bacteria have evolved ribosome rescue pathways. The tmRNA–SmpB system is found in nearly all sequenced bacteria and utilizes SmpB and the *ssrA* tmRNA, a small RNA with properties similar to both tRNA and mRNA. When translating transcripts without a stop codon, ribosomes stall at the 3′ end of the mRNA. This stalling recruits *ssrA* RNA, which is translated into a tag at the C-terminus of the defective peptide. Cellular proteases then degrade this tagged peptide, and the ribosome is freed after translation of this tagged peptide [40–42] (Figure 1D).

**Figure 2:**
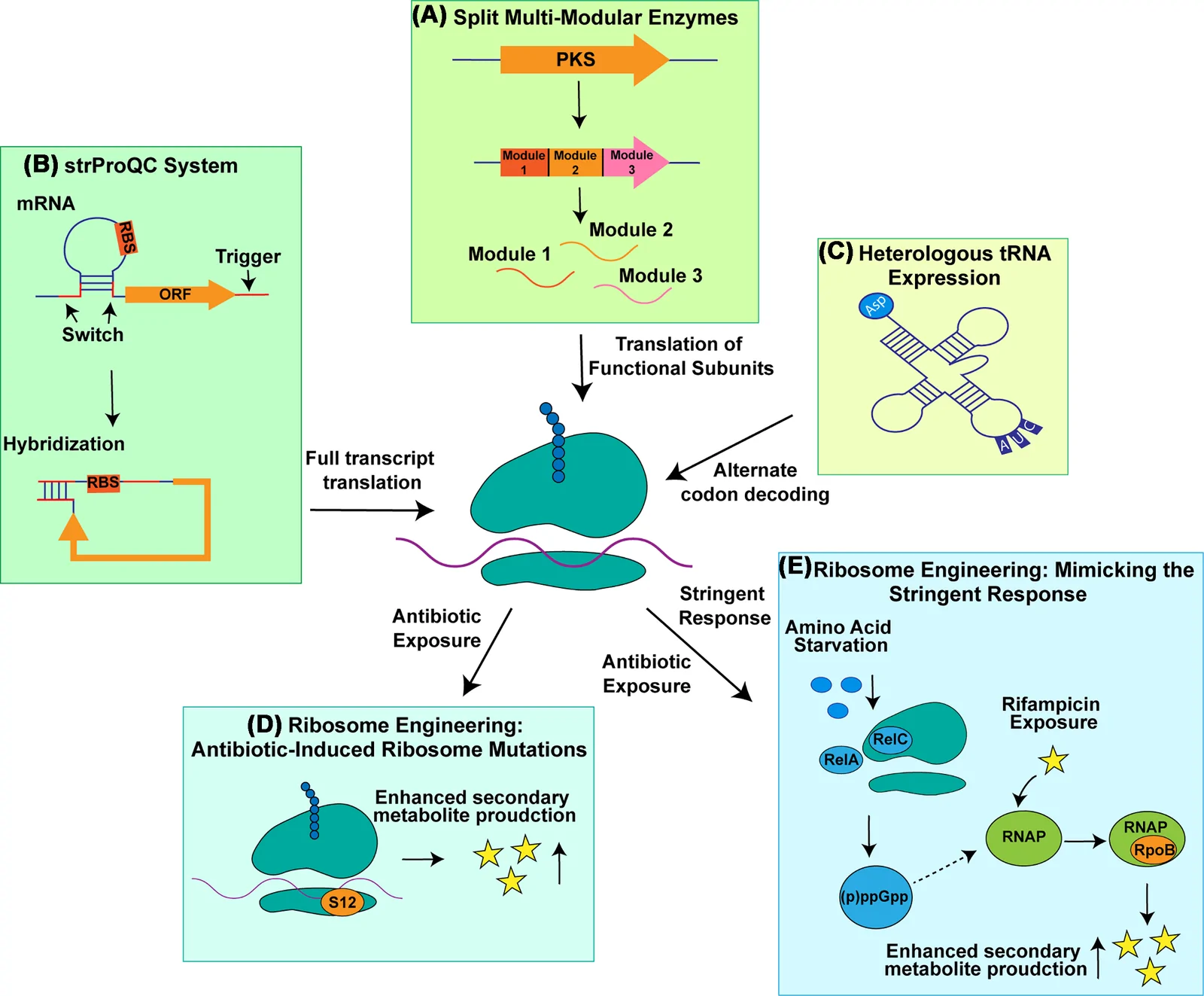
Translational engineering strategies to stimulate secondary metabolite expression (**A**) Splitting multi-modular biosynthetic enzymes into functional subunits increases translation efficiency by reducing the amount of truncated mRNAs and the need for ribosome rescue. (**B**) The *Streptomyces* protein quality (strProQC) system hybridizes the 3′ and 5′ ends of mRNA using a switch RNA, encapsulating the RBS within a secondary structure and a trigger RNA. This hybridization stabilizes long biosynthetic transcripts and increases translational efficiency, which enhances biosynthetic enzyme translation. (**C**) Heterologous expression of tRNA^Asp^_AUC_ circumvents wobble base pairing to enhance translation efficiency and BGC expression (top right). (**D**) Antibiotic-induced mutations in ribosomal proteins also lead to the activation or enhancement of secondary metabolism. (**E**) RNA polymerase mutations induced by the antibiotic rifampicin mimic (p)ppGpp-bound states and stimulate the production of antibiotics.

PKSs are enzymes essential for the biosynthesis of many valuable antibiotics, antitumor drugs, and immunosuppressants [43–45]. Because these modular enzymes are large and prone to transcript truncation, recent studies aim to develop systems to improve the translation of functional biosynthetic enzymes. *Streptomyces albus* J1074 encodes genes for the biosynthesis of the polyketide butenyl-spinosyn, a highly potent insecticide. The three-module PKS gene involved in biosynthesis of butenyl-spinosyn, *busA*, is exceptionally large at 13 kb. Analysis of transcripts associated with *busA* revealed that greater than 93% of transcripts are truncated [46]. Overexpression of the *busA* gene leads to up-regulation of the tmRNA–SmpB system, therefore stalling ribosomes. To improve the translation efficiency of long PKS transcripts, Liu et al. split *busA* into three functional biosynthetic genes that are separately translated by ribosomes, which reduced the need for ribosome rescue and improved translation efficiency (Figure 2A). Similar results were observed with other large PKS genes including *aveA2*, the 18.7-kb ORF in the avermectin BGC, and *epoD*, the 21.8-kb ORF in the epothilone BGC [46]. Therefore, splitting large multi-modular enzymes into functional subunits can serve as a new strategy to improve translation efficiency of large biosynthetic genes.

Eukaryotes have evolved a system to prevent the translation of truncated transcripts and promote translation of full-length mRNA [47,48]. A similar system has been applied to improve translation efficiency in *E. coli* known as the protein quality control (ProQC) system. The ProQC system has two components. One is a switch RNA that encapsulates the start codon and RBS within a secondary structure. The other is a trigger RNA complementary to the switch RNA at the 3′ end of a transcript. The trigger RNA hybridizes with the switch RNA promoting transcript circularization and exposing the translation initiation site to the ribosome [49]. Recently, this system has been adapted for use in *Streptomyces* to ensure that only full-length transcripts occupy translating ribosomes. Jiang et al. developed and applied the strProQC system to enhance translation of full-length PKS transcripts [39] (Figure 2B). This system was employed to assist in functional expression of large PKS genes in *S. albus* for the 7.8-kb *spnA* gene for spinosad biosynthesis and in *S. avermitilis* for the 25.7-kb *rapA* gene for rapamycin biosynthesis. In both cases, application of the strProQC enhanced metabolite expression by 1.4-fold and 4.7-fold, respectively, compared with streptomycetes lacking the system. Thus, engineering translational quality control provides a powerful strategy for enhancement of natural products biosynthesized by large multi-modular enzymes.

## The impact of codon usage and tRNA abundance on translation efficiency

Transfer RNAs are essential mediators of translation required for decoding transcripts and delivering amino acids to the ribosome. Thus, codon usage and tRNA availability profoundly influence protein synthesis. The *bldA* locus in the *S. coelicolor* genome encodes the sole tRNA for decoding the rare leucine codon, UUA, which is exceptionally uncommon in high GC organisms like streptomycetes. Only approximately 2%–3% of coding sequences contain the TTA codons [50]. The TTA codon is only found in genes involved in secondary metabolism and is not present in any primary metabolic genes in the model streptomycete [51,52]. Although the *bldA* locus is nonessential, mutants exhibit a ‘bald’ phenotype characterized by a lack of aerial hyphae and the inability to produce pigments and antibiotics. This phenotype is attributed to its inability to translate genes containing TTA codons. The methylenomycin BGC in *S. coelicolor* contains TTA codons in *mmfL* and *mmyB*, a biosynthetic gene involved in production of the signaling molecule gamma-butyrolactone and a transcriptional activator, respectively [53]. Both genes are essential for activation of the biosynthesis of methylenomycin. In bald *S. coelicolor* mutants deficient in antibiotic production, replacing both rare leucine codons with the alternative common CTC restores methylenomycin production [54]. In contrast, other antibiotics that do not have TTA codons in their biosynthetic genes are also no longer expressed in bald mutants, including actinorhodin and undecylprodigiosin, likely due to regulatory defects beyond the BGC [55].

Bald mutants experience a widespread antibiotic deficit, thus implying that TTA codons are present in global regulators. The global transcriptional regulator AdpA is responsible for regulation of growth morphology and production of antibiotics in streptomycetes. In many species, expression of AdpA requires tRNA^Leu^_UUA_ for its decoding [55–57]. AdpA positively regulates biosynthetic genes involved in streptomycin biosynthesis in *Streptomyces griseus* [58], clavulanic acid biosynthesis in *Streptomyces clavuligerus* [59], and chloramphenicol biosynthesis in *S. venezuelae* [60]. Therefore, use of the UUA codon, and abundance of its cognate tRNA, has been suggested to be a regulatory switch for secondary metabolism at the translation level [61]. However, the idea of this tRNA acting as a regulator is controversial because there is evidence of expression of TTA-containing genes in the absence of *bldA*. Bioinformatic analysis suggests that downstream nucleotides can impact mistranslation of NNA codons; however, no special case has been identified to explain this phenomenon in genes containing TTA codons [61]. Additional studies on *bldA* and TTA-codon dependence will shed light on the evolution and function of this important translational control of secondary metabolism.

Nevertheless, manipulation of tRNA pools is a promising strategy for enhancing natural product levels in streptomycetes. Chen et al. discovered a novel tRNA from a desert streptomycete, *Streptomyces violaceusniger* strain SPC6, that decodes aspartate GAU codons. This tRNA^Asp^_AUC_ contributes to decoding GAU codons and potentially GAC aspartate codons (Figure 2C). The heterologous expression of this tRNA in other streptomycetes increases translational efficiency through circumvention of inefficient wobble base pairing [62]. Crick’s wobble hypothesis explains that a single tRNA can decode multiple codons through a noncanonical pairing between the third nucleotide in a mRNA codon and the first nucleotide in the tRNA anticodon [63]. Queuosine is a tRNA modification in the wobble base position of tRNA anticodons for decoding GUN codons to improve translation efficiency. Heterologous expression of tRNA^Asp^_AUC_ in *S. coelicolor* enhanced production of all antibiotics, and expression of this tRNA enhanced secondary metabolite production in all streptomycetes tested [62]. These data highlight the importance of tRNA pools in expression of biosynthetic genes and exhibit how the manipulation of tRNA pools can stimulate secondary metabolism.

## Ribosome engineering: antibiotic-induced mutations stimulate secondary metabolism

While modulation of tRNA pools can influence decoding ability, the ribosome itself is a major point of translational control. Alterations to the ribosome can dramatically impact secondary metabolism. Spontaneous mutations in ribosomal proteins conferring resistance to ribosome-targeting antibiotics stimulate the biosynthesis of secondary metabolites. Across several streptomycete species, resistance mutations in *rpsL* ribosomal subunit S12 enhance secondary metabolism. Notably, the K88E mutation confers resistance to streptomycin and is one of the most extensively studied mutations for its ability to stimulate secondary metabolism [64]. Mutations in *rpsL* trigger an overproduction of several antibiotics, including actinorhodin in *S. coelicolor* and *Streptomyces lividans* [64], actinomycin in *S. antibioticus* and *Streptomyces parvulus*, and oligomycin in *S. avermitilis* [65]. Similarly, inactivation of *rsmG*, a 16S rRNA methyltransferase, confers resistance to streptomycin and results in increased protein synthesis activity during late growth phase [66–68]. The *rsmG* null mutations enhance actinorhodin production in *S. coelicolor*, actinomycin production in *S. antibioticus* and *S. parvulus*, and moderately increase oligomycin production in *S. avermitilis*. Additionally, point mutations that substitute Lys43, Lys45, Arg86, and Lys88 of RsmG enhance antibiotic production [65]. Together, these observations highlight the value of exploiting ribosomal mutations for enhanced secondary metabolite production, with many additional mutations and mechanisms beyond those described here having been extensively studied across streptomycete species (Figures 1F and 2D).

While direct mutations in ribosomal proteins impact secondary metabolism, the global regulatory stringent response indirectly impacts translational activity in response to nutrient-lacking conditions. The bacterial stringent response is active when amino acid deficiencies trigger utilization of guanosine 5′-diphosphate 3′-diphosphate (ppGpp) and guanosine 5′-triphosphate 3′-diphosphate (pppGpp) alarmones for regulation of global gene expression and protein synthesis. Amino acid depletion is detected when uncharged tRNAs bind to the ribosome, triggering (p)ppGpp synthesis. Production of (p)ppGpp is dependent on the *relA* ppGpp synthetase gene and the *relC* 50S ribosomal subunit protein L11 [69,70]. Studies with mutations in *relA* and *relC* suggest that (p)ppGpp alarmones may trigger antibiotic production in streptomycetes. The repression of (p)ppGpp through *relC* mutations in *S. antibioticus* reduces expression and transcript levels of genes involved in the biosynthesis of actinomycin [64,71]. The presumed mechanism for reducing expression of biosynthetic genes in cells lacking (p)ppGpp is through binding to RNA polymerase and inhibiting the transcription of genes for translation, up-regulating amino acid biosynthesis pathways, and conferring resistance to antibiotics targeting RNA polymerase. Through binding of (p)ppGpp, BGCs that are silent under standard nutrient-rich conditions are expressed during amino acid starvation [70]. Mutations in the *rpoB* mimic this (p)ppGpp-bound state of RNA polymerase and confer resistance to the transcription inhibitor rifampicin [11,72] (Figure 2E). Therefore, utilization of antibiotics to modify translational machinery, both directly and as a downstream effect, is a useful strategy for improved expression of biosynthetic genes.

## ABCF induction of BGC expression in response to antibiotics

ATP-binding cassette family F ATPases (ABCFs) are a class of ribosome-modulating ATPases that have recently received attention for their housekeeping and ARE functions. ABCFs are widespread among all kingdoms except archaea, and bacteria have on average four ABCFs within their genomes [73]. The *Actinobacteria* phylum in particular has 7 specific subfamilies of ABCF proteins [74] and species within the phylum encode up to 11 ABCF proteins per genome [75]. These proteins are classified as ‘housekeeping’ proteins that rescue ribosomes stalled at difficult to translate motifs or as ARE ABCFs that rescue ribosomes stalled by antibiotics. Housekeeping ABCFs, such as the well-studied EttA in *E. coli*, are global regulators that stabilize the ribosome or rescue stalling when stretches of basic or acidic residues or polyprolines challenge translation efficiency, impact the proteome, and influence metabolic flux [76–78]. Similarly, ARE ABCFs rescue antibiotic-stalled ribosomes through either inducing a conformational change in the ribosome, causing the antibiotic to dissociate, or through displacement of the drug from the ribosome [79,80] (Figure 1E).

New studies suggest that ARE ABCFs can also act as regulators that activate antibiotic production in concentration-dependent manners [74,81]. The ARE ABCF LmrC belongs to the ARE5 subfamily conferring resistance to LS_A_P antibiotics and is encoded in the lincomycin BGC of *S. lincolnensis*. LmrC not only provides self-resistance to lincomycin but also stimulates lincomycin biosynthesis [74]. The lincomycin BGC encodes three resistance proteins: LmrA, LmrB, and LmrC [82], raising the question of why the organism requires this redundancy. Only LmrA, a transporter, and LmrB, a 23 rRNA methyltransferase, are required for lincomycin resistance. LmrC plays a key role in a signal cascade, stimulating the activation of the transcriptional regulator LmbU [74,83,84] in tandem with LmrA and LmrB, which negatively affect this activation [74]. LmrU activates the 4-alkyl-L-proline biosynthetic gene required for lincomycin production [74,83,85]. Additional regulation for full lincomycin BGC expression is regulated by AdpA_lin_ [86]. Therefore, the LmrC-dependent signal cascade likely ensures uniform expression of lincomycin biosynthetic genes within a population.

Unlike LmrC, TiaA and Are5sc are two ARE5 ABCFs found outside BGCs in *S. coelicolor*, but nonetheless orchestrate dose-dependent BGC activation and regulate the global antibiotic response [81]. Because these are not coded within a BGC, their function was previously unclear. Koberska et al. recently elucidated the ARE functions of both TiaA and Are5sc. TiaA rescues ribosomes targeted by tiamulin and pleuromutilin, the latter being an antibiotic produced by fungi that *S. coelicolor* may encounter in its natural environment. Are5sc appears to be a weak ARE factor itself but likely works in tandem with TiaA to provide resistance to LS_A_P antibiotics [81]. Prior studies showed that actinorhodin production in *S. coelicolor* is triggered by ribosome stress through an unknown mechanism [87,88]. The present study attributes activation of the actinorhodin BGC to dose-dependent regulation by ABCF proteins in response to LS_A_P antibiotics [81].

Are5sc and TiaA are additionally able to distinguish between functionally similar ribosome-targeting antibiotics to provide differential effects on gene expression [81]. The amount of actinorhodin produced by *S. coelicolor* varies by LS_A_P exposure, with the greatest expression in response to lincomycin. In addition to inducing actinorhodin BCG expression, TiaA impacts expression of global secondary metabolism. At high subinhibitory (8 mg/l) concentrations of tiamulin, TiaA suppresses the production of antibiotics by *S. coelicolor* [81]. Therefore, ARE ABCF proteins can double as global regulators controlling secondary metabolism and responses to antibiotic exposure in *Streptomyces* at the translation level. Future studies should explore the function of uncharacterized ABCF proteins across streptomycetes and the manipulation of ARE ABCF proteins in streptomycetes to stimulate natural product biosynthesis in a similar manner to the well-studied ribosome engineering.

## Conclusions

The integrated control of translation is gaining recognition as a key determinant of secondary metabolism and a promising target for enhancing natural product yields. At the translation ‘hub,’ many contributors, including regulatory RNAs, RNases, transcript quality control, tRNA pools, ribosomal alterations, and ribosome rescue factors, converge to maintain tight control of protein abundance, also known as translational buffering. The present essay has highlighted mechanisms where translation modifications impact BGC expression. The examples presented suggest that there is more to learn through the study of translational control in streptomycetes and their impact on secondary metabolite expression. In addition to the well-studied ribosomal engineering to enhance natural product expression, recent studies have explored modifications of other factors involved in protein synthesis that also improve translation efficiency and stimulate secondary metabolism (Figure 2 and Table 1). Studies on translation mechanisms open the possibility for novel strain engineering strategies. Therefore, knowledge of these mechanisms may greatly improve strain engineering and antibiotic discovery in the future.

**Table 1:** Descriptions of major translation regulatory mechanisms impacting secondary metabolism in *Streptomyces* spp.

Regulatory mechanism	Description
Small regulatory RNAs (sRNAs)	Noncoding RNAs influencing transcript stability and regulating the translational efficiency of transcripts.
Cis-antisense RNAs (asRNAs)	A type of noncoding RNA encoded on the complementary strand.
Convergent and untranslated RNAs (cutoRNAs)	A type of asRNA present at the overlap of a 3′UTR and a convergently encoded gene.
5′ untranslated region (5′UTR)	Untranslated region upstream of open reading frames, the length of which affects translation efficiency.
RNase ES	RNase E ortholog suppressing secondary metabolism through transcript degradation.
RNase III	A double-strand-specific endonuclease influencing secondary metabolism and rRNA maturation.
RNase J	A 5′ to 3′ exonuclease regulating secondary metabolism and rRNA maturation.
tmRNA–SmpB system	A common translation rescue system found across bacterial species that rescues ribosomes stalled on truncated transcripts.
*Streptomyces* protein quality control system (strProQC)	A system previously applied to *E. coli* now adapted in streptomycetes to promote the translation of full-length PKS transcripts through transcript circularization.
tRNA^Leu^_UUA_	tRNA for the translation of the rare leucine codon TTA encoded at the *bldA* locus.
tRNA^Asp^_AUC_	tRNA from *Streptomyces violaceusniger* heterologous expression improving translational efficiency of GAU codons through circumvention of wobble base pairing.
Ribosome engineering	Antibiotic resistance mutations that alter ribosomal and RNA polymerase proteins stimulate secondary metabolite production.
ATP-binding cassette family F ATPases (ABCFs)	ATPases that rescue ribosomes stalled by antibiotics or nascent peptides with unfavorable geometries. Recent studies show ABCFs activate BGC expression in response to antibiotic exposure.

## Summary

- Understanding the regulation at the translation level is important for engineering the expression of bioactive secondary metabolites.
- Transcripts occupying ribosomes are more tightly controlled than transcript abundance; therefore, translation efficiency is important in global regulation.
- ncRNAs, RNases, transcript quality control, tRNA pools, codon usage, antibiotic-induced ribosomal mutations, and ribosome rescue mechanisms impact expression of secondary metabolic genes.
- Manipulation of translation components can be applied as strategies to engineer streptomycetes for enhanced natural product production.

## Open Access

Open access for this article was enabled by the participation of Texas A&M University in an all-inclusive Read & Publish agreement with Portland Press and the Biochemical Society.

## Abbreviations

3′UTR: 3′ untranslated region
5′UTR: 5′ untranslated region
ABCF: ATP-binding cassette family F ATPase
ARE: antibiotic resistance
asRNA: cis-antisense RNA
BGC: biosynthetic gene cluster
cutoRNA: convergent and untranslated RNA
LS_A_P: lincosamides, streptogramin A, and pleuromutilin
ncRNA: Noncoding RNA
ORF: open reading frame
PKSs: polyketide synthases
ppGpp: guanosine 5′-diphosphate 3′-diphosphate
pppGpp: guanosine 5′-triphosphate 3′-diphospate
ProQC: protein quality control system
RBS: ribosome binding site
RNase: ribonuclease
SmpB: small protein B
sRNA: small regulatory RNA
strProQC: *Streptomyces* protein quality control
tmRNA: transfer messenger RNA
